# The economic costs of malaria in children in three sub-Saharan countries: Ghana, Tanzania and Kenya

**DOI:** 10.1186/1475-2875-12-307

**Published:** 2013-09-03

**Authors:** Elisa Sicuri, Ana Vieta, Leandro Lindner, Dagna Constenla, Christophe Sauboin

**Affiliations:** 1 Barcelona Centre for International Health Research (CRESIB, Hospital Clínic-Universitat de Barcelona), Barcelona, Spain; 2 CIBER Epidemiología y Salud Pública (CIBERESP), Barcelona, Spain; 3 Health Economics and Outcome Research - IMS Health, Barcelona, Spain; 4 Johns Hopkins Bloomberg School of Public Health, Baltimore, Maryland, USA; 5 Health Economics, GlaxoSmithKline Vaccines, Wavre, Belgium

**Keywords:** Malaria, Sub-Saharan Africa, Costs

## Abstract

**Background:**

Malaria causes significant mortality and morbidity in sub-Saharan Africa (SSA), especially among children less than five years of age (U5 children). Although the economic burden of malaria in this region has been assessed previously, the extent and variation of this burden remains unclear. This study aimed to estimate the economic costs of malaria in U5 children in three countries (Ghana, Tanzania and Kenya).

**Methods:**

Health system and household costs previously estimated were integrated with costs associated with co-morbidities, complications and productivity losses due to death. Several models were developed to estimate the expected treatment cost per episode per child, across different age groups, by level of severity and with or without controlling for treatment-seeking behaviour. Total annual costs (2009) were calculated by multiplying the treatment cost per episode according to severity by the number of episodes. Annual health system prevention costs were added to this estimate.

**Results:**

Household and health system costs per malaria episode ranged from approximately US$ 5 for non-complicated malaria in Tanzania to US$ 288 for cerebral malaria with neurological sequelae in Kenya. On average, up to 55% of these costs in Ghana and Tanzania and 70% in Kenya were assumed by the household, and of these costs 46% in Ghana and 85% in Tanzania and Kenya were indirect costs. Expected values of potential future earnings (in thousands) lost due to premature death of children aged 0–1 and 1–4 years were US$ 11.8 and US$ 13.8 in Ghana, US$ 6.9 and US$ 8.1 in Tanzania, and US$ 7.6 and US$ 8.9 in Kenya, respectively. The expected treatment costs per episode per child ranged from a minimum of US$ 1.29 for children aged 2–11 months in Tanzania to a maximum of US$ 22.9 for children aged 0–24 months in Kenya. The total annual costs (in millions) were estimated at US$ 37.8, US$ 131.9 and US$ 109.0 nationwide in Ghana, Tanzania and Kenya and included average treatment costs per case of US$ 11.99, US$ 6.79 and US$ 20.54, respectively.

**Conclusion:**

This study provides important insight into the economic burden of malaria in SSA that may assist policy makers when designing future malaria control interventions.

## Background

Despite a declining trend in the number of cases and deaths over the last few years, malaria still causes significant mortality and morbidity worldwide [1]. According to the World Health Organization (WHO), approximately 225 million cases of malaria were estimated to have occurred worldwide in 2009, leading to 781,000 deaths. Ninety one percent (~709,000 deaths) of these deaths occurred in the African region [1]. The bulk of the burden of malaria is observed in children under five years of age (U5 children). In sub-Saharan Africa (SSA), the severity of the disease in this age group is evident, with malaria attacks leading to one million cases of cerebral malaria and four million cases of severe anemia each year. Among the children with clinical attacks of malaria, several thousand were estimated to have experienced neurological damage and up to 250,000 have developmental problems [2].

In the context of increasing attention towards improved malaria control in settings with budget constraints, competing health problems and weak health systems [3], it is essential to provide policy makers with relevant economic evidence of the economic benefits of health care control and prevention strategies under different conditions and scenarios [4,5]. This information can guide the introduction of new preventative measures, improve current strategies for malaria control and help to design the scaling up of both new and old efficacious interventions.

Previous cost studies have reported the economic burden of malaria to households and to the health system in SSA, but the extent and variation of this impact remains unclear. Household costs for malaria treatment, in particular indirect costs, are not adequately explored due to difficulties in collecting and estimating these data [6-8]. Furthermore, while a lot of attention has focused on both household and health system costs of uncomplicated malaria, there is limited knowledge of the economic impact of severe cases, its consequences and co-morbidities [9].

Various factors affect the economic burden of malaria among children in endemic areas, including: treatment-seeking behaviour [10], age of child [11] and epidemiological conditions [12]. Despite this complexity, studies of the economic burden of malaria generally estimate average costs or cost distributions for an episode and the effects of epidemiological, behavioural or clinical factors are rarely explored in association with costs [9].

The overall aim of this study was to estimate the economic burden of malaria in U5 from the household and health system perspectives in three selected SSA countries. Specific objectives were to estimate: (i) the costs of treatment per malaria episode by severity and presence of co-morbidities and clinical complications; (ii) the expected treatment cost per episode per child; (iii) and third, the annual economic costs of malaria, including both prevention and treatment costs.

## Methods

Three countries were selected to provide estimates for different epidemiological settings within the SSA region: Ghana, Tanzania and Kenya. Ghana is a West African country with about 23 million inhabitants, presenting high malaria endemicity, with 100% of the population living in high transmission areas. Tanzania, with almost 42 million inhabitants including Zanzibar, is an East African country with moderate malaria endemicity. Almost three quarters (73%) of the population in Tanzania live in high transmission areas and approximately one quarter in areas of low transmission. Kenya is another East African country with relatively low malaria endemicity. Kenya has more than 39 million inhabitants with 36% of the population living in high transmission areas, 40% in low transmission areas, and 24% in malaria-free zones [13].

### Cost estimates

The costs of treating uncomplicated (outpatients without co-morbidities) and hospitalized cases (all cases requiring parenteral treatment, despite WHO case definitions for severe malaria) were based in each of the three countries on earlier studies that evaluated the economics of intermittent preventive treatment of malaria in infants (IPTi) and in children (IPTc) [14,15]. In IPTi study, data were collected at different health facilities representing the three levels of health care i.e. primary, secondary and tertiary care in each country. Household costs were collected through surveys from a sample of carers of approximately 300 children after an outpatient visit or at discharge (150 outpatients and 150 inpatients) in Kenya and Tanzania. Data collection in Ghana included 207 outpatients and 10 inpatients cases interviewed at home [15]. Household costs were divided into direct and indirect. Direct costs were then divided into the cost of the visit or hospitalization (including facilities and personnel) and the cost of the resources used for treatment (tests and medications). Indirect costs included the carers’ reported productivity loss for the entire episode of malaria. Health provider treatment costs included both recurrent and capital costs attributable to malaria care in U5 children.

The breakdown of costs collected during the IPTi study were updated to 2009 rates using the Consumer Price Index of the USA [16] and combined treatment costs for co-morbidities, such as anemia, cerebral malaria and neurological sequelae. In addition, costs were modified according to the new first-line treatment for uncomplicated malaria introduced in recent years [artemisinin-based combination therapy (ACT)]. ACT costs incurred by the households were taken from a recent report [17]. International drug supplier prices were used and augmented by 15% to include shipment costs when drug costs were entirely borne by the health system [18].

Standards of care and associated costs of co-morbidities and complications were estimated based on interviews with clinicians, health workers and managers of the malaria control programme in the three countries.

In the current study, incremental costs associated with treatment and care of co-morbidities and medium-term consequences not included in the base estimates were considered, for both the health system and the household. Health system additional costs were considered in terms of incremental personnel effort and other resources, such as the extra costs associated with the administration of parenteral treatment compared to oral therapies. Household additional costs were considered in terms of incremental direct (user fees, transportation) and indirect costs (additional value of time lost). Drug costs were imputed to the health system or households depending on national or local policies. Specific costs associated with severity of disease and the presence of co-morbidities was: cost of blood transfusion (severe anemia), cost of anti-seizure/anticonvulsant therapies (cerebral malaria) and rehabilitation costs post-discharge (neurological sequelae).

A live chicken was assumed to be the payment for traditional treatment for one episode of malaria [19,20]. Institutional local market prices were used to estimate the monetary value of such a payment [21]. Total costs for treating a malaria episode were estimated for the following categories: uncomplicated malaria, malaria hospitalization, malaria hospitalization + severe anemia, cerebral malaria, and cerebral malaria + neurological sequelae. Malaria hospitalization refers to all inpatient cases, regardless of being severe cases according to WHO definition. These categories were based on the perceptions of clinicians and health workers interviewed rather than on institutional definitions. ‘*Uncomplicated malaria*’ included all malaria cases (usually laboratory confirmed) where no hospitalization was required. ‘*Cerebral malaria*’ was generally referred to as malaria hospitalization of children in deep coma. Total costs were calculated by adding health system and household costs and subtracting user fees paid by the households for consultation or admission at health facility.

The human capital approach was applied to estimate the potential life-long productivity losses due to death. This cost was represented, in each country, by the present value of an annuity with instalment equal to the institutional minimum wage in force, for the period defined by adulthood (from 15 years) and life expectancy [22,23]. The present value at the time of childhood death, of future potential earnings for an individual (onset of work at 15 years of age) was calculated using the following formula:

$$V=R\left\{\left[1-{\left(1+i\right)}^{-n}\right]/i\right\}*{\left(1+i\right)}^{-m}$$

*R* is the annual earning; *n* is the time (in years) between 15 years and life expectancy; *i* is the discount rate (assumed to be 3%); *m* represents the number of years between childhood death and 15 years of age. In the model, death was assumed to occur either at 0–1 or 1–4 years of age. Life expectancies differ between these two age groups [24].

### Description of the models

Models were developed to estimate the expected treatment cost per malaria episode per child by severity and presence of co-morbidities and clinical complications, from the household and health system perspectives. Therefore, the result of each model is the expected value of treatment cost per episode per child, including household and health system costs.

**Figure 1:**
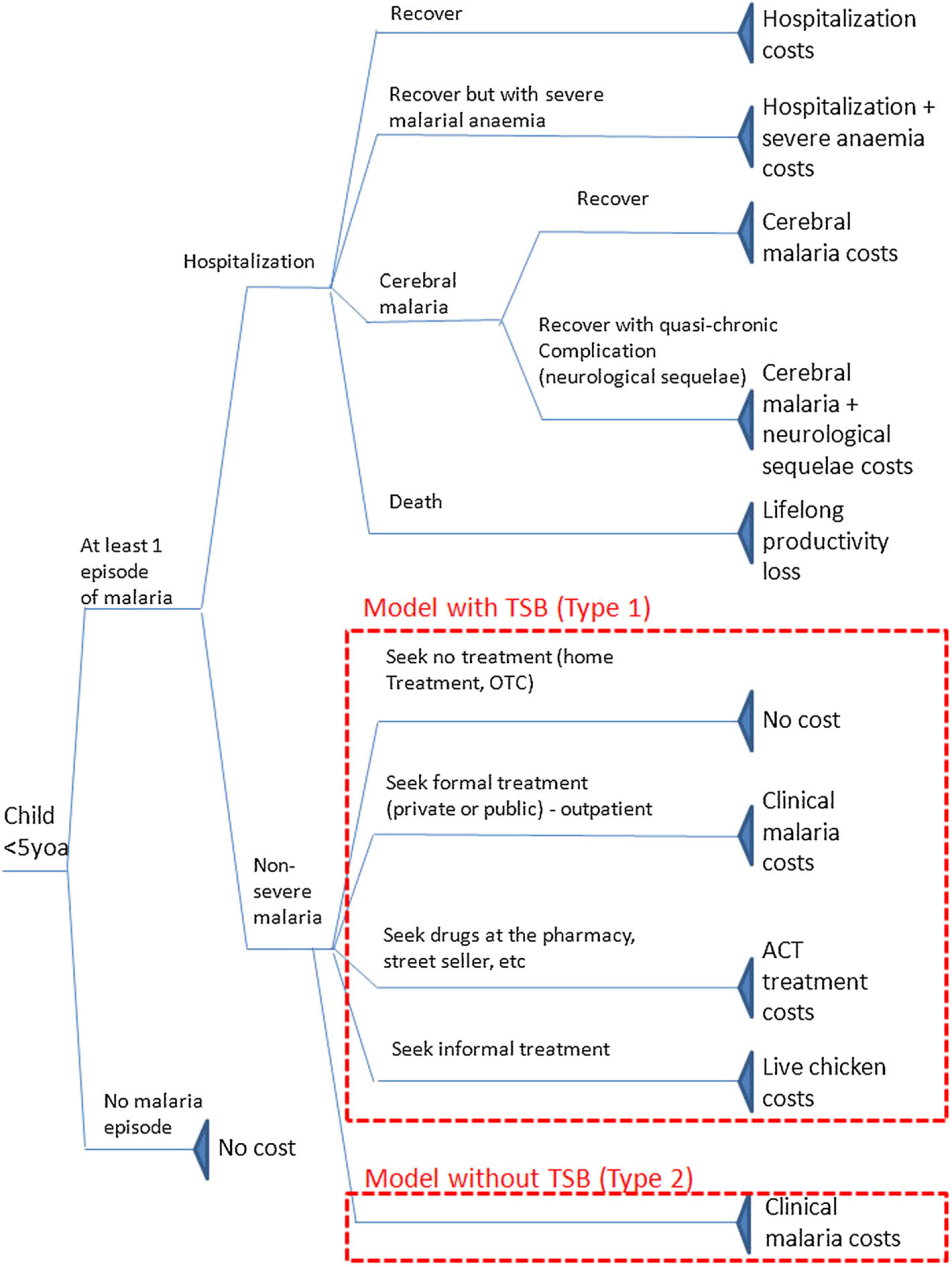
Models to estimate expected malaria treatment costs per episode per child (U5) and with or without treatment seeking behaviour (TSB) component.

Probabilities of incurring a malaria episode were taken from the results of previous clinical trials (Table 1) where health outcomes were measured at health facilities rather than within the community. Therefore, such data may be biased towards more intensive users of health services. In order to assess if treatment-seeking behaviour would impact the results, two different types of models were constructed. The two models are identical in structure except that the treatment-seeking behaviour for uncomplicated malaria was considered in model type 1 but not in model type 2 (Figure 1). The models start with the probability of experiencing at least one episode of malaria, consider the probability of such an episode becoming severe and conclude with the probability of the severe episodes (with and without co-morbidities) resulting in sequelae or death. In model type 1, episodes of non-complicated malaria are associated with different types of costs depending on the type of treatment sought. No cost was applied when malaria treatment was not sought.

**Table 1:** Model inputs and main sources

***Country and age range***	***Clinical/Epidemiological data***						***Treatment-seeking behaviour***			
	**UM**	**MO**	**SA**	CerM*	CerM + NS*	**CFR**	**No treatment**	**Health facility**	**Pharmacy/ shop**	**Traditional treatment**
**Ghana**										
2-15 months	0.64-1 [25]	0.06 [25]	0.006 [25]	0.015 [25]	0.12-0.15	0.0141 [25]	0.3	0.545	0.15	0.005
16-24 months	0.33-0.65 [25]	0.03 [25]	0.003 [25]	0.02 [25]	0.12-0.15	0.0157 [25]				
0-24 months	0.65-1 [26]	0.05 [27]	0.03 [26]	0.022-0.08 [28,29]	0.12-0.15	0.003**				
3-18 months	0.84-1 [27]	0.05 [27]	0.44 [27]	0.022-0.08 [28,29]	0.12-0.15	0.003**				
3-59 months	0.51-0.63 [30]	0.004 [30]	0.02 [30]	0.022-0.08 [28,29]	0.12-0.15	0.003**				
**Tanzania**										
2-12 months	0.49 [31]	0.04 [32]	0.13 [31]	0.022-0.08 [28,29]	0.12-0.15	0.0051**	0.26	0.62	0.1167	0.005
2-11 months	0.16-0.24 [32]	0.04 [32]	0.25 [32]	0.022-0.08 [28,29]	0.12-0.15	0.0051**				
12-23 months	0.39-0.50 [32]	0.05 [32]	0.24 [32]	0.022-0.08 [28,29]	0.12-0.15	0.0051**				
0-59 months	0.28 [33]	0.05 [32]	0.24 [32]	0.022-0.08 [28,29]	0.12-0.15	0.0051**				
**Kenya**										
0-12 months	0.14-1 [34]	0.21 [34]	0.23 [34]	0.022-0.08 [28,29]	0.12-0.15	0.0053**	0.32	0.45	0.224	0.0086
10-24 months	0.17-1 [34]	0.21 [34]	0.23 [34]	0.022-0.08 [28,29]	0.12-0.15	0.0053**				
0-24 months	0.67-0.86 [35]	0.21 [34]	0.26 [35]	0.022-0.08 [28,29]	0.12-0.15	0.0053**				

Notes: All clinical/epidemiological values represent probabilities. *UM* uncomplicated malaria, *MO* malaria hospitalization, *SA* severe anaemia, *CerM* cerebral malaria, *CerM + NS* cerebral malaria and neurological sequelae, *CFR* case fatality rate: percentage of clinical cases with fatal outcome. *CerM represents the proportion of hospitalizations clinically considered as cerebral malaria while CerM + NS the proportion of CerM with neurological sequelae. **Most CFR were calculated as average ratios among number of malaria deaths in U5 children/number of malaria cases in U5 children taken from World Malaria Reports (from year 2000 up to 2009). Triangular distributions were used when only one value is presented (assuming a range of 15% lower and higher). Uniform distributions were used where two values are presented. Ranges for severe anemia were constructed based on Murphy *et al.*[36]. Main data sources on neurological sequelae were taken from Bassat *et al.*[28]. References for treatment-seeking behaviour are Demographic and Health surveys of each of the three countries [37-39]. Values are the percentage of people who sought treatment among whom with fever in the two weeks previous the survey.

### Scenarios

The burden of malaria in children varies by age. Therefore, several versions of the two model types were developed by age groups for which clinical incidence data were available [40,41]. In order to account for different epidemiological contexts, the models were used for cost estimation in Ghana, Tanzania and Kenya separately. To represent intra-country epidemiological heterogeneity, data from different areas were used. The two types of model were estimated for five age groups in Ghana, three age groups in Kenya and four age groups in Tanzania. Therefore, a total of 24 scenarios were constructed.

### Model inputs and sources

Table 1 summarizes parameters used to populate the models (Figure 1), with their relative sources. The age-specific probabilities of experiencing at least one episode of malaria, the probability of hospitalization and of co-morbidities or complications/sequelae were taken from several different sources, including clinical trials for IPTi and of Intermittent Preventive Treatment of malaria in children (IPTc) [25-27,30-32,34]. Probabilities included in the models were obtained by rates published as outcomes of children in the control group of each trial considered, translated into yearly probabilities [42]. Case fatality rates (CFR) were calculated as average ratios of the number of malaria deaths in U5 children to the number of malaria cases in U5 children reported in World Malaria Reports (between 2000 and 2009), apart from the case of Ghana (2–15 and 16–24 months children) for which malaria death was included among trial outcomes [25].

Information on treatment-seeking behaviour for malaria was taken from Demographic and Health Survey (DHS) data bases (Standard DHS, Standard AIS – AIDS indicator surveys - in the case of Tanzania) [37-39]. For model type 1, Chi-square tests were performed to test the presence of a statistically significant association between the age of the child and treatment choices.

### Sensitivity analyses

Most of the variables used to populate the models were taken from studies providing local information, with both cost and epidemiological data being derived from small rural areas in each country (Table 1, [14]). To test the uncertainty around estimated mean values, sensitivity analyses were conducted. More specifically, input variables were assigned a range of possible values, to generate a probability distribution. Triangular or uniform distributions were constructed with the estimates of the current study being the most likely value, and the minimum or the maximum being used as the comparator value.

Values used as comparators for health system costs for malaria treatment were WHO-choice cost estimates [43]. For each country, comparator household costs for malaria treatment were derived from different sources. For Ghana, household costs, comparator costs were taken from Asante *et al.*[44]. Household costs in Tanzania were taken from Hutton *et al.*[45]. For Kenya, estimates from Chuma *et al.*[46] were used as comparators for uncomplicated case costs; Ayieko *et a*l. [47] cost data were used to estimate the costs of complicated cases.

Epidemiological data were also inserted as a probability distribution by comparing estimates with values taken from the World Malaria Report 2009 [13]. Monte Carlo simulations were performed within the constructed ranges, (N = 1000 iterations). All analyses were performed using TreeAge Software. 2008. TreeAge Pro 2008 (Tree Age Software, Inc., Williamstown, MA, USA).

### Annual cost estimates

As no age breakdown is reported in the World Malaria Report 2010 [1], the total number of malaria cases occurring in U5 children during the year 2009 in Ghana and Tanzania was estimated by assuming the same proportion between U5 cases and all-age cases published in the World Malaria Report 2009 [13]. For Kenya, the number of malaria cases occurring in U5 children was assumed to be 40% of cases occurring across all ages [48]. U5 malaria cases were grouped according to severity, using the same clinical/epidemiological data mentioned above (Table 1). Each unit cost per episode, for households and the health system, was multiplied by the number of cases grouped by severity. Death was included in the household indirect cost calculation and the value of one death was represented by the net present value of future potential earnings. Total annual costs were presented from both the households and the health system perspectives. The proportion attributable to U5 children of total annual costs for prevention, from the health system perspective (bed nets and indoor residual spraying) and for the same year in each country, was added to household and health system costs to yield total annual costs [49,50]. The proportion of prevention costs imputable to U5 children was calculated by multiplying the total cost by the proportion of the total population accounted for U5 children in each country (28%, 18% and 17% for Ghana, Tanzania and Kenya, respectively [51]). Average treatment costs, including household and health system, were calculated by dividing total costs (excluding prevention) by the total number of cases.

## Results

### Costs per episode based on severity

**Figure 2:**
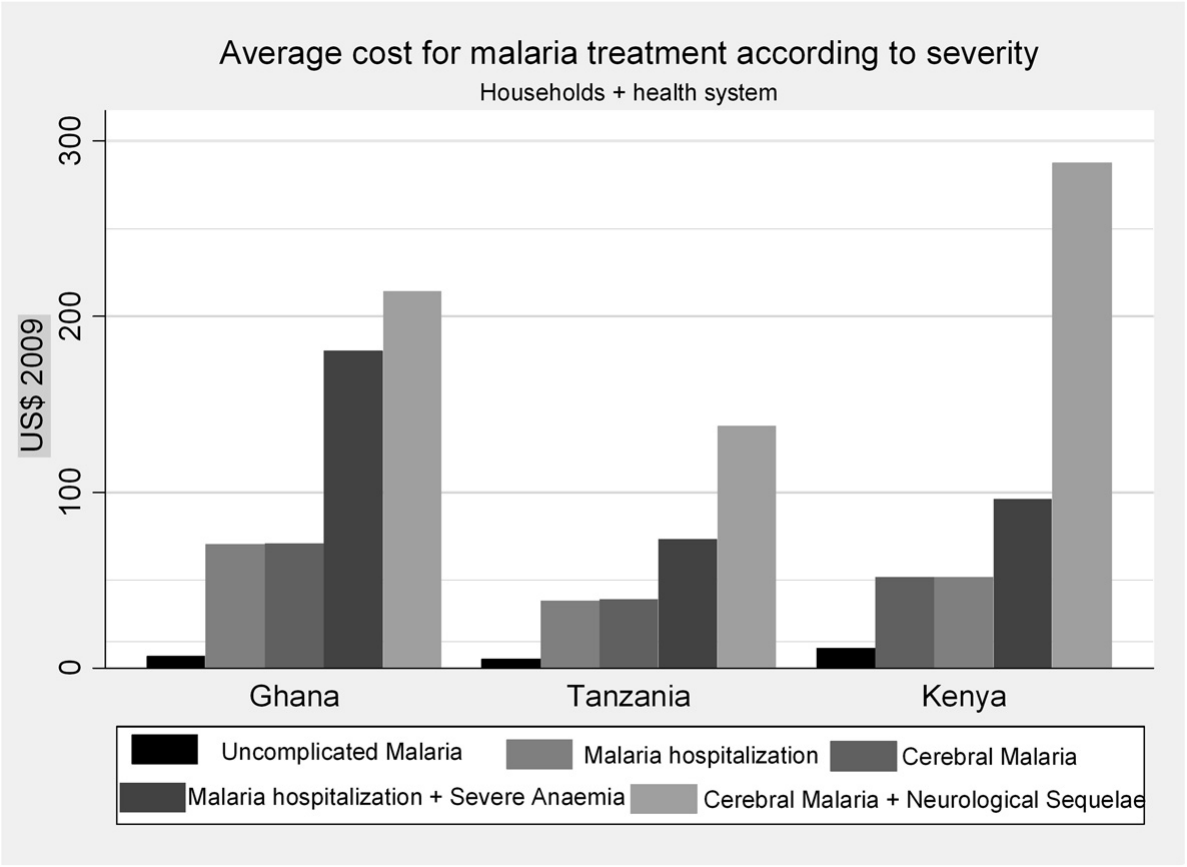
Total treatment costs according to severity.

Standards of care by country and the breakdown costs based on severity are summarized in Tables 2 and 3, respectively. Total costs per malaria episode (including direct and indirect household costs and health system costs) based on disease severity and the presence of complication and co-morbidities ranged from US$ 7.99 to $ 229.24 in Ghana, from US$ 5.2 to $ 137.74 in Tanzania, and from US$ 11.24 to $ 287.81 in Kenya (Table 3, Figure 2).

**Table 2:** Summary of standards of care by country (year 2009)

**Malaria severity**	**Antimalarial drugs**	**Other drugs (non-anti-malarial)**	**Average length of stay**	**Other treatments**
**Ghana**				
**UM**	Artesunate-amodiaquine (80%); artemether-lumefantrine (20%)	Paracetamol (100%); promethazine (20%)	0	-
**MO**	Quinine (100%)	Paracetamol (100%); phenobarbital (10%)	7	-
**MO + SA**	Quinine (100%)	Gentamicin (10%); cotrimoxazole (10%)	10	Iron supplementation (100%); blood transfusion (50%)
**CerM**	Quinine (80%); artemether lumefantrine (20%)	Paracetamol (80%); hydrocortison (5%); phenobarbital (50%)	7	
**CerM + NS**	Quinine (80%); artemether lumefantrine (20%)	Paracetamol (80%); hydrocortison (5%); phenobarbital (50%)	12	Rehabilitation visit every 10 days for 1 year
**Tanzania**				
**UM**	Artesunate-Amodiaquine (75%); Quinine (25%)	Paracetamol (100%); promethazine (20%)	0	-
**MO**	Quinine (100%)	Paracetamol (100%)	7	-
**MO + SA**	Quinine (100%)	Gentamicin (10%); cotrimoxazole (10%)	7	Iron supplementation (100%); blood transfusion (50%)
**CerM**	Quinine (100%)	Paracetamol (60%); phenobarbital (30%)	7	
**CerM + NS**	Quinine (100%)	Paracetamol (60%); phenobarbital (30%)	10	Rehabilitation visit every 15 days for 1 year
**Kenya**				
**UM**	Artesunate-amodiaquine (75%); artemether-lumefantrine (25%)	Paracetamol (100%); promethazine (20%)	0	-
**MO**	Quinine (80%); artemether lumefantrine (20%)	Paracetamol (100%); phenobarbital (10%)	7	-
**MO + SA**	Quinine (80%); artemether lumefantrine (20%)	Gentamicin (10%); cotrimoxazole (10%)	9	Iron supplementation (100%); blood transfusion (50%)
**CerM**	Quinine (80%); artemether lumefantrine (20%)	Paracetamol (70%); phenobarbital (30%)	7	
**CerM + NS**	Quinine (80%); artemether lumefantrine (20%)	Paracetamol (70%); hydrocortison (5%); phenobarbital (30%)	10	Rehabilitation visit every 15 days for 1 year

Notes: *UM* uncomplicated malaria, *MO* malaria hospitalization, *SA* severe anemia, *CerM* cerebral malaria, *CerM + NS* cerebral malaria and neurological sequelae. In brackets, the proportion of cases receiving treatment.

**Table 3:** Total cost of one episode of malaria by severity in 2009 US$

	**Household**			Health system total* costs	
**Malaria case**	**Direct**	**Indirect**	**Total**		
**Ghana**					
**UM**	4.44	1.26	5.70	2.89	*7.99*
**MO**	24.51	24.22	48.73	27.49	*75.62*
**MO + SA**	74.67	53.50	128.17	64.1	*191.67*
**CerM**	24.51	24.66	49.17	27.49	*76.06*
**CerM + NS**	36.75	70.20	106.95	122.89	*229.24*
***Average***	*32.98*	*34.77*	*67.74*	*48.97*	*116.12*
**Tanzania**					
**UM**	0.42	3.14	3.56	1.75	*5.2*
**MO**	5.46	14.36	19.82	18.56	*38.38*
**MO + SA**	5.46	20	25.46	48.21	*73.67*
**CerM**	5.46	14.36	19.82	19.13	*38.95*
**CerM + NS**	7.86	89.72	97.58	40.16	*137.74*
***Average***	*4.93*	*28.32*	*33.25*	*25.56*	*58.79*
**Kenya**					
**UM**	0.73	7.95	8.68	2.77	*11.24*
**MO**	10.88	21.4	32.28	20.86	*51.89*
**MO + SA**	10.88	35	45.88	54.26	*96.57*
**CerM**	10.88	21.4	32.28	20.86	*51.89*
**CerM + NS**	20	212.2	232.2	56.86	*287.81*
***Average***	*10.67*	*59.59*	*70.26*	*31.12*	*99.88*

Notes: *UM* uncomplicated malaria, *MO* malaria hospitalization, *SA* severe anemia, *CerM* cerebral malaria, *CerM + NS* cerebral malaria and neurological sequelae. *Total treatment costs = household direct + household indirect + health system – user fees.

### Cost incurred by households

On average, up to 55% of the overall costs in Ghana and Tanzania and 70% in Kenya were borne by households. Most of household costs are made of indirect costs (85%) in Tanzania and in Kenya while this proportion falls to about half (46%) in Ghana. This pattern repeats over different severity levels. In Ghana, households need to support much more direct costs than in the two other countries of the study due to more expensive treatment and higher medical service costs, which include high health personnel salary. In the case of cerebral malaria with neurological sequelae households in Kenya have the highest economic burden with large productivity losses.

Average indirect costs are higher in Kenya with close to US$ 8 per uncomplicated episode. This figure falls to US$ 1.26 in Ghana where the lower minimum wage in force brings the productivity losses down. Unsurprisingly indirect costs increase sharply for hospitalized cases and cases with neurological sequelae for which follow-up is required leading to the highest costs: US$ 70, US$ 90 and US$ 212 in Ghana, Tanzania and Kenya respectively (Table 3).

### Cost incurred by the health system

Health system costs per episode vary according to severity from US$ 2.89 to US$ 123 in Ghana, US$ 1.75 to US$ 48 in Tanzania and US$ 2.77 to US$ 57 in Kenya. Regardless of severity, Ghana health system costs are higher than for the other two countries due to higher personnel costs. Costs of hospitalized cases are about tenfold the cost of uncomplicated case and they increase substantially when severe anemia or neurological sequelae occurs. Blood transfusion is an expensive procedure used in the first case while frequent follow-up visits are necessary in the latter case. Average treatment costs and length of stay were similar for malaria-related hospitalization with or without cerebral consequences.

### Models

Across the three countries, there was no statistical significance observed in the association between the age of the child and the choice of treatment (χ^2^-p value > 0.05). For this reason, the same proportion of non-severe cases seeking treatment was assumed in the different treatment options regardless of age.

In addition to treatment costs per episode of malaria according to severity (Table 3), the price of a live chicken (US$ 3.79 in Ghana, US$ 4.94 in Tanzania and US$ 5.70 in Kenya) was used as a proxy for traditional treatment cost and the cost of ACT was used when seeking care at the pharmacy [17]. To calculate potential productivity loss due to death for age ranging from 0–1 and 1–4 years, age-specific life expectancy values were used. Productivity losses differed amongst the three countries and were US$ 11,794 and US$ 13,814 in Ghana, US$ 6,856 and US$ 8,066 in Tanzania, and US$ 7,554 and US$ 8,897 in Kenya (Table 4). The higher figures for Ghana are due to a longer life expectancy.

**Table 4:** Other costs included in the models

**Type of cost**		**Life expectancy**	**2009 US$**
**Ghana**			
Productivity loss due to infant deaths*		56.6	11794.01
Potential productivity loss due to children death**		60.2	13814.85
Traditional treatment^+^		-	3.79
ACT median costs (median, interquartile range)		-	1.37 [0.68, 2.40]
**Tanzania**			
Productivity loss due to infant deaths*		50.3	6856.98
Potential productivity loss due to child death**		53.3	8066.92
Traditional treatment^+^		-	4.94
ACT median costs (median, interquartile range)		-	0.70 [0.35, 2.15]
**Kenya**			
Productivity loss due to infant deaths*		53.5	7554.08
Potential productivity loss due to child death**		57.1	8897.63
Traditional treatment^+^		-	5.70
ACT median costs (median, interquartile range)		-	0.66 [0.53, 1.71]

Notes: *0-1 year; ** 1-4 years; ^+^as a proxy, the cost of a live chicken was used; *ACT* Artemisinin Combination Therapy.

Results of the Monte Carlo simulations (Table 5) showed that the mean expected malaria treatment cost per episode per child ranged from US$ 5.45 to US$ 22.3 in Ghana, US$ 1.29 to US$ 5.47 in Tanzania, and US$ 13.57 to US$ 22.88 in Kenya.

**Table 5:** Expected treatment cost of a malaria episode by age-group and model type (Monte Carlo simulation)

	**Expected treatment cost (US$) 2009**									
Country and age range*	Model type 1 with TSB**					Model type 2 without TSB**				
	***Mean***	***Confidence interval***		***Min***	***Max***	***Mean***	***Confidence interval***		***Min***	***Max***
				**Ghana**						
2-15 months	22.3	11.7	40.48	8.11	54.16	20.42	14.38	28.38	12.45	32.71
3-24 months	10.65	7.2	14.77	5.78	16.85	15.25	10.43	21.43	8.75	25.93
0-24 months	15.31	8.35	26.32	6.31	36.83	16.79	10.5	25.64	8.42	36.09
16-24 months	8.34	5.26	12.48	4.2	14.22	10.94	2.58	16.52	5.49	19.17
3-59 months	5.45	3.26	8.38	2.7	10.45	8.47	5.27	12.74	4.69	15.24
				**Tanzania**						
2-12 months	3.49	2.62	4.48	2.37	4.94	4.15	3.77	4.59	3.66	4.68
2-11 months	1.29	1.01	1.59	0.93	1.7	1.66	1.32	2.06	1.2	2.6
12-23 months	3.41	2.84	4	2.61	4.31	4.26	3.64	4.98	3.4	5.4
0-59 months	2.12	1.9	2.36	1.8	2.47	5.47	3.45	7.58	2.78	8.07
				**Kenya**						
0-12 months	13.57	3.88	24.01	3.27	25.20	15.69	4.40	27.60	3.51	29.49
10-24 months	15.23	5.05	25.62	4.30	27.20	17.37	5.62	29.41	4.66	31.35
0-24 months	19.90	17.19	22.74	16.18	23.69	22.88	19.43	26.49	18.01	27.60

Notes: *References of input values used in Table 1; **TSB treatment-seeking behaviour.

Finally, total annual economic costs for treatment and prevention of malaria in U5 children for the year 2009 were (in millions) US$ 37.8 in Ghana; US$ 131.98 in Tanzania; and US$ 109.04 in Kenya (Table 6). Of these costs, household treatment costs were (in millions) US$ 5.98, US$ 17.98 and US$ 45.23 for the three countries, respectively. Prevention costs were (in millions) US$ 29, US$ 104 and US$ 42, respectively. When considering productivity loss due to premature death, these figures increased to (in millions) US$ 66.97, US$ 290.57 and US$ 250.71, respectively. The average cost of a malaria episode was estimated to reach US$ 12 in Ghana, US$ 6.8 in Tanzania and US$ 20.5 in Kenya when weighing unit costs with the occurrences for each outcome but leaving out the prevention costs and mortality-related productivity loss (Table 6).

**Table 6:** Annual economic costs (US$) for malaria treatment and prevention (for 2009)

	**Number of cases**^**a**^	**Household cost per episode**^**b**^	**Health system cost per episode**^**c**^	**Total household costs**	**Total health system treatment cost**	**Total cost**	**Average treatment cost per case**
			**Ghana**				
Uncomplicated malaria	721,827	5.70	2.89	*4,114,412*	*2,086,079*	*6.200,492*	
Malaria hospitalization	36,091	48.73	27.49	*1,758,731*	*992,151*	*2,750,882*	
Cerebral malaria	1,643	49.17	27.49	*80,792*	*45,169*	*125,960*	
Sequelae	256	106.95	122.89	*27,426*	*31,514*	*58,940*	
Death	2,279	12,804.43		*29,187,094*		*29,187,094*	
Health system prevention costs						*28,643,462*	
*TOTAL including costs associated with productivity loss due to death*				*35,168,455*		*66,966,829*	
*TOTAL excluding costs associated with productivity loss death*				*5,981,361*	*3,154,913*	*37,779,735*	11.99
			**Tanzania**				
Uncomplicated malaria	3,979,828	3.56	1.75	*14,168,187*	*6,964,699*	*21,132,886*	
Malaria hospitalization	178,155	19.82	18.56	*3,531,024*	*3,306,549*	*6,837,573*	
Cerebral malaria	8,111	19.82	19.13	*160,755*	*155,158*	*315,913*	
Sequelae	1,266	97.58	40.16	*123,520*	*50,836*	*174,356*	
Death	21,254	7,461.95		*158,592,785*		*158,592,785*	
Health system prevention costs						*103,520,000*	
*TOTAL including costs associated with productivity loss due to death*				*176,576,271*		*290,573,513*	
*TOTAL excluding costs associated with productivity loss death*				*17,983,486*	*10,477,242*	*131,980,728*	6.79
			**Kenya**				
Uncomplicated malaria	2,567,086	8.68	2.77	*22,282,304*	*7,110,827*	*29,393,132*	
Malaria hospitalization	648,270	32.28	20.86	*20,926,168*	*13,522,920*	*34,449,088*	
Cerebral malaria	29,513	32.28	20.86	*952,691*	*615,649*	*1,568,340*	
Sequelae	4,606	232.20	56.86	*1,069,544*	*261,905*	*1,331,448*	
Death	17,222	8,225.85		*141,667,404*		*141,667,404*	
Health system prevention costs						*42,300,000*	
*TOTAL including costs associated with productivity loss due to death*				*186,898,111*		*250,709,412*	
*TOTAL excluding costs associated with productivity loss death*				*45,230,707*	*21,511,301*	*109,042,008*	20.54

Notes: ^a^The total number of malaria cases occurring in U5 children in 2009 was estimated for each country by assuming the same proportion of total cases accounted for by U5 cases as published in the World Malaria Report 2010; ^b, c^see Table 3.

## Discussion

In the current study, the economic costs of malaria in three SSA countries with different levels of malaria endemicity were investigated. Costs associated with malaria in U5 children in Ghana, Tanzania and Kenya was substantial, particularly when severity of the episode, co-morbidities and medium term consequences were considered. National annual costs for the prevention and treatment of malaria in U5 children corresponded to 0.14%, 0.62% and 0.36% of the Gross Domestic Product (GDP) (year 2009) of Ghana, Tanzania and Kenya, respectively [52].

It is expected that higher investments in prevention should lead to lower treatment cost, however, the relationship between the two variables is not usually straightforward. Both prevention and treatment costs were included in national annual costs of malaria to represent the total cost for malaria. However, no consideration was applied on their relationship, as this goes beyond the scope of this study.

There were slight differences in the treatment of severe and cerebral malaria [28]. In Ghana treatment was different from Kenya and Tanzania for the use of hydrocortisone in 5% of cases classified as cerebral malaria.

When combining the cost for each severity level with their probability of occurrence, the type 1 model estimated lower costs than type 2 model. The first model considers indeed a proportion of uncomplicated cases not seeking treatment, therefore not generating any cost. For each of the three countries the percentage of cases of fever with no treatment was around 30% and consequently a cost equal to zero had a strong impact on the average total cost for malaria. The exception is Ghana (2–15 months) where the price paid for ACT at the pharmacy (U$ 1.37 on average, much higher than costs incurred at public health facilities) has increased the expected cost in model type 1.

Previously published data for Ghana reported the average costs for health care provider treatment and for households (direct and indirect) to be around US$ 6.87 and US$ 15.79, respectively [44]. These are lower than the estimates in the current study. Previous data from 2009 showed the mean cost of a malaria admission in Kenya, from the provider perspective, was higher than that estimated in the current study (US$ 95.58 vs. US$ 20.86 or US$ 54.26 if severe anemia is a co-morbidity) [47]. In Tanzania, health provider costs were similar to those estimated in this study, with previous estimates of US$ 20.0 for the treatment of one episode of malaria (combining outpatient and inpatient cases) and US$ 22.3 for one episode of severe anemia [53]. However, care must be taken when comparing estimates from different studies, since in this study, productivity loss due to premature death was also considered and looked at a variety of severity levels. Although direct comparisons with other studies are not straightforward due to different objectives and to different methodologies, this study used previous estimates as comparator values in the sensitivity analysis. This allowed us to control for uncertainty of parameters used and for the potential intra-country variability of costs [54].

From the health system and the household perspective, cases of severe malarial anemia incurred higher costs than cases of cerebral malaria with no severe anemia. These higher costs were due to blood transfusions. Among other costs, the administration of blood transfusions increases personnel costs. Personnel costs are also higher for cases of cerebral malaria that result in neurological sequelae, due to the rehabilitation/follow up of the children. From the household perspective, the cost of an episode of cerebral malaria with neurological complications represented 30% of the GDP per capita in Kenya [55]. For each of the three countries, direct household costs depend on whether households incur the cost of drug treatment. One paradox with drug treatment is that these costs are greater for uncomplicated malaria than for severe malaria because ACT, which is used for the treatment of uncomplicated malaria, is more expensive than quinine, which was the first-line treatment for severe cases at the time of the study.

The method used for assessing productivity losses due to premature death, the human capital approach, although widely used has some limitations [22,55]. For instance, this approach ignores other dimensions of illness (such as pain and suffering) as well as non-market activities (such as loss of leisure) that may be as important to individuals as economic loss. Another issue that needs attention involves the choice of an appropriate social discount rate to convert future earnings into present values. Discount rate depends on risk aversion and on time preferences: the use of acceptable values for these parameters would imply further investigation that was beyond the aim of this study. Therefore, these specific productivity losses were reported separately.

The estimated productivity losses are substantial, especially considering that the GDP per capita in each of the three countries studied is about US$ 2.0 per day [52]. Potential productivity losses were higher for older than for younger children because of their longer life expectancy. From an epidemiological point of view however, the probability of being infected is higher in younger than in older children. Results of the models highlighted some compensation effect between these two factors.

Looking at expected costs per child, in the case of Ghana, data could be easily compared between children aged 2–15 months and children aged 16–24 months, as epidemiological data were taken from the same clinical trial. The higher productivity losses of older children were compensated by the lower probability of infection, which was equal to 0.64-1 for children aged 2–15 months and 0.33-0.65 for children aged 16–24 months, as the CFR is very similar across the two age groups [25]. For Kenya, a straightforward cost comparison could be performed between children within the age ranges 0–12 months and 10–24 months, as data from these age groups were taken from the same clinical trial [34]. In contrast to Ghana, expected malaria costs were higher for older children (10–24 months) than for younger children (0–12 months). In Kenya expected costs were higher when children were followed up 0–24 months due to the high probability of both clinical and severe symptomatic malaria occurring during the clinical study [35]. It is important to point out that variation across age groups within the same country pick up also intra-country epidemiological heterogeneity as nearly all information for the different age groups were taken from different clinical trials.

Net present value of lifelong productivity losses was used to represent the cost of premature patient death. However, the major short-term cost following the death of a person in SSA is the funeral. There is strong evidence, at least for South Africa, to suggest that funeral expenses have a substantial impact on household budgets. In a recent study it was found that, on average, households spend the equivalent of one year’s income for a funeral for an adult [56].

During the last few years a national scheme of health insurance has been introduced in Ghana [57]. In this study, a scenario was considered in which no one had health insurance and everyone had to pay for health care in Ghana. However, it has been shown that health insurance cardholders accounted for 7% of the population in 2005 and 45% of the population in 2008 [58]. Despite this increase in health insurance coverage, the assumption that households need to pay for health care may only have a minor effect on results, as was reported in a recent study that health insurance in Ghana had a positive and significant impact on utilization of health care services but had no effect on out-of-pocket expenditure [59]. Moreover, in Tanzania and Kenya health insurance schemes exist, the adhesion to which is compulsory, respectively, for public servants and for all salaried employees. However, the coverage of such schemes is low in both countries and the impact on results should be minor [60,61].

Results reported may underestimate true costs, especially for costs paid by the households, due to various factors. First, funeral costs were not considered because a child’s funeral cost is unknown and there is no evidence to suggest that out-of-pocket expenses for funeral of a child are the same as for an adult. Second, treatment costs incurred by households may have also been underestimated. There is evidence of “informal payments” being used to facilitate access to health care. Studies have shown that, when informal payments are considered, the total cost for malaria treatment may be three times higher than that reported by health care providers [62-64]. Third, the cost of traditional treatment may be far higher than the proxy used in this study [65]. Fourth, annual household costs did not include household prevention costs. These were not collected during the IPTi study as surveys were undertaken for children with malaria, which can be an important source of bias for estimation of prevention costs. Fifth, although costs associated with severe anemia were estimated and used in the models, as a conservative measure, they were not included in the aggregate annual costs of malaria due to the complex and unclear interaction between malaria and anemia [66]. A further source of underestimation may derive from diagnostic costs, included in health system costs. As diagnostic cost estimates were included in clinical studies, these all refer to blood smear which may have a low incremental impact on total costs.

On the other hand, a source of overestimation of costs may be the assumption that children with uncomplicated malaria received ACT only at health facilities or at private pharmacies. Although ACT was adopted as first-line its actual level of use remains low [67]. The assumption was necessary due to the absence of precise estimates of access to ACT for the countries included in this study. The WHO recently modified their recommendation, to include ACT for treating severe malaria cases as well as uncomplicated malaria. This is expected to increase treatment costs.

## Conclusions

Malaria exerts a significant economic burden on health care providers and households. Cost estimations for a single malaria episode provide important information. However, it is fundamental to consider severity, co-morbidities and, at least, medium-term consequences when estimating the economic burden of malaria. Additional insights are provided by examining how costs vary as a function of factors that affect the probability of incurring an infection. The results of this study may help to guide the introduction of new prophylactic measures, to improve current strategies for malaria control and to design the scaling up of both new and old efficacious interventions.

## Abbreviations

AIS: AIDS indicator survey; ACT: Artemisinin-based combination therapy; CFR: case fatality rates; DHS: Demographic and Health survey; GDP: Gross domestic product; IPTc: Intermittent preventive treatment of malaria in children; IPTi: Intermittent preventive treatment of malaria in infants; SSA: sub-Saharan Africa; U5: children below five years of age; USA: United States of America; WHO: World Health Organization.

## Competing interests

AV and LL were consultants at IMS; ES was health economists at CRESIB and all three were paid through a contract research project financed by GlaxoSmithKline group of companies to conduct the study. DC was an employee of GlaxoSmithKline group of companies at the time the study was conducted and CS is an employee of GlaxoSmithKline group of companies and owns stock options.

## Authors’ contributions

ES conceived the study, gathered the information, analysed the data, and wrote the manuscript. AV provided substantial contribution to study design and results interpretation. DC provided substantial scientific input to the study, assisting in the conception and design of the study, developing the methodology, checking results robustness, and revising drafts of the manuscript critically for important intellectual content. LL collaborated in the conception of the study, fieldwork, gathered the information, data analysis, and in the revision of the manuscript. LL provided substantial scientific input to the study, assisting in the conception and design of the study, developing the methodology, checking results robustness, and revising drafts of the manuscript critically for important intellectual content. CS checked and gathered information, reviewed the methodology, analysed data, contributed to the manuscript writing and reviewed the full content. All authors read and approved the final manuscript.
